# Perks of the Pandemic?: The Impact of the COVID-19 Pandemic on Socially Withdrawn Emerging Adults

**DOI:** 10.1177/21676968231184283

**Published:** 2023-06-26

**Authors:** Mallory A. Millett, Larry J. Nelson, William J. Burk, Yvonne H. M. van den Berg

**Affiliations:** 1 Behavioural Science Institute, 6029 Radboud University Nijmegen, Nijmegen, Netherlands; 2 School of Family Life, 6756 Brigham Young University, Provo, UT, USA

**Keywords:** social withdrawal, emerging adulthood, COVID-19, identity, mental health

## Abstract

Emerging adults were among those especially affected by the distancing measures and instability brought on by the COVID-19 pandemic. However, not all emerging adults were affected equally. Thus, the purpose of this paper was to determine if different types of socially withdrawn emerging adults (shy, unsocial, avoidant, mixed-withdrawn) were affected differently from one another and from non-withdrawn emerging adults. Pandemic impact was measured in terms of changes in mental distress, life satisfaction, and identity development from before the pandemic to during the pandemic. Participants were 1249 emerging adults from project READY, a nationally representative, longitudinal study of young adults in the United States. Results showed that mixed-withdrawn emerging adults decreased in mental distress from before the pandemic to during the pandemic while non-withdrawn emerging adults increased in mental distress. Additionally, there was a decrease in life satisfaction across groups, and no significant change in identity development across time.

## Introduction

Since the beginning of the global pandemic, nearly everyone has been impacted in some way by COVID-19 and the subsequent measures set in motion to control the spread of the virus. Though emerging adults generally experienced fewer pandemic related health risks compared to other age groups, recent research suggests that they may have been impacted the most by the distancing measures and instability brought on by the spread of COVID-19 (Daly et al., 2020; Pierce et al., 2020; Reyes-Portillo et al., 2022; Varma et al., 2021). However, not all emerging adults seem to be equally affected and certain cultural and individual characteristics may act as buffers against pandemic related stress (Germani et al., 2020; Padilla-Walker et al., 2022; van den Berg et al., 2021). One individual characteristic of interest during a time when social contact is limited is a person’s pre-pandemic tendency towards social withdrawal, or in other words, the degree to which a person tends to *choose* to withdraw from social interactions. The purpose of this study was therefore to examine whether the impact of the COVID-19 pandemic on emerging adults’ well-being and development varied as a function of their personal tendency to socially withdraw.

### Social Withdrawal in Emerging Adulthood

Social withdrawal is an “umbrella construct” that describes the behavioral tendency to avoid or withdraw from social interactions (Rubin et al., 2009). Based on Asendorpf’s model of social motivations (1990), socially withdrawn individuals can be divided into three specific subtypes based on their motivations to approach and avoid social situations. The first subtype is *shyness* which is characterized by both a high approach and a high avoidance motivation. In other words, shy individuals typically want to interact socially but also feel the desire to avoid social gatherings out of fear or wariness. The second subtype called *unsociability*, is characterized by both a low approach and a low avoidance motivation. These individuals typically can participate socially if they choose, but they simply prefer solitude over social interaction. Finally, the third subtype of withdrawal called *avoidance*, is characterized by a low approach motivation and a high avoidance motivation. These individuals seem to dislike social interaction and avoid it whenever possible.

These three subtypes of withdrawal are not only unique in their conceptualization, but also in their correlates with adjustment and maladjustment during emerging adulthood. For example, the tension between approach and avoidance motivations means that shyness is often associated with mental distress (Nelson et al., 2008, 2015), difficulties with relationships (Nelson, 2013), and a delay in the transition to adult roles (Asendorpf et al., 2008; Kerr et al., 1996). In contrast, unsociability seems to be relatively benign (Coplan et al., 2019; Nelson, 2013) and may even provide some benefits, specifically with regards to creativity (Bowker et al., 2017) and the formation of one’s identity (Barry et al., 2013). Finally, avoidance is related to a number of negative outcomes, like internalizing problems, poorer quality relationships (Nelson, 2013), problematic media use (Nelson et al., 2016), and externalizing behaviors (Clifford et al., 2022). Though much less is understood about avoidance than the other two subtypes, researchers have suggested that these individuals may actively choose to avoid social interaction because they have experienced high levels of peer rejection and exclusion in the past (Bowker & Raja, 2011). Based on these differences in underlying motivations as well as the distinct correlates with mental health and well-being, it can be expected that not all socially withdrawn individuals may have been equally affected by the COVID-19 pandemic.

### Social Withdrawal in Emerging Adulthood during COVID-19

When we think of the unique social circumstances (or lack of social circumstances) created by COVID-19 lockdowns and social distancing regulations, the subject of social withdrawal becomes particularly interesting. Indeed, Asendorpf’s model (1990) relies heavily on motivations to approach and avoid social situations, but what happens when there is no longer a need to avoid socializing or there are no social situations to approach? It may be that those with high avoidance motivations experience some relief over not having to put themselves in situations that make them uncomfortable, while those with high approach motivations experience extra stress at having their social interactions limited. In other words, emerging adults who were actively seeking solitude over social interaction before the pandemic may not have been equally affected by distancing regulations as highly social emerging adults. Additionally, because of said distancing regulations, the pandemic has also provided the unique opportunity to examine how broader changes in societal expectations about sociability may generally affect the well-being of socially withdrawn individuals. Thus, understanding the impact of the pandemic on socially withdrawn emerging adults becomes relevant beyond even the circumstances of the current pandemic.

#### Mental Distress and Life Satisfaction

There are several reasons to believe that COVID-19 had differential impact on emerging adults’ mental distress and life satisfaction. A recent meta-analysis of longitudinal studies examining the impact of COVID-19 on mental health showed that there was a general increase in symptoms of mental illness during the initial peak of the pandemic, but that these symptoms returned to pre-pandemic levels after a few months (Robinson et al., 2022). However, these studies predominantly looked at the impact of COVID-19 on mental illnesses in the first few months of the pandemic. We also know little about the impact of the pandemic after the summer of 2020, when new waves of the virus emerged and distancing measures were reintroduced. Moreover, the majority of these longitudinal studies use broad samples of adults, and we know much less about the specific impact over time on younger generations, like emerging adults (Chadi et al., 2022). Nevertheless, recent studies have shown that emerging adults reported the highest levels of pandemic related mental distress out of any age group during the first few months of the pandemic (Pierce et al., 2020; Varma et al., 2021). Indeed, because emerging adulthood is a time of instability, young adults may have been particularly susceptible to increased distress when faced with the academic disruptions and loneliness brought on by the pandemic (Haikalis et al., 2021; Reyes-Portillo et al., 2022). Additionally, many emerging adults reported they were generally less satisfied with their life during the COVID-19 pandemic than before (Ammar et al., 2020; Preetz et al., 2021).

With regards to the impact of the pandemic on the mental health and life satisfaction of socially withdrawn individuals in particular, there is not a lot of literature. However, it stands to reason that differing motivations for approaching and avoiding social interactions before the pandemic would lead to a difference in pandemic related impact. For example, at a time when avoiding social interaction is socially acceptable and even encouraged, avoidant individuals may experience some relief at not having to socialize and, therefore, experience decreases in mental distress and increases in life satisfaction. Indeed, a study examining the well-being of socially withdrawn adults in China during the first months of the pandemic found an increase in well-being for avoidant individuals (Xu et al., 2022). In contrast, this study found that shy adults remained stable in their well-being. This may be because shy individuals have a high approach motivation in addition to their high avoidance motivation. On the one hand, the change from large, in-person events to smaller-scale, online interactions may gratify shy emerging adults’ avoidance motivation (Dikaya et al., 2021). Yet, on the other hand, shy emerging adults are known to rely heavily on the support of friends to be able to socialize (Closson et al., 2019). Thus, the distance from others created by the pandemic may have made it especially hard for shy emerging adults to meet their social needs. Overall, the added benefits and challenges of the pandemic may balance each other out for shy individuals leading to more stable levels of mental distress and life satisfaction.

Unsocial emerging adults do not have a strong need to approach or avoid social interaction and thus, we might expect their mental health and life satisfaction to be unaffected by the social changes of the pandemic. Yet, the same study of Chinese adults showed a temporary decrease in well-being for unsocial adults during the first months of the pandemic that returned to normal by the summer of 2020 (Xu et al., 2022). This indicates that even those with low approach motivations may have been impacted by social distancing. However, this may be due to the severity of the social distancing measures in China whereas the impact for unsocial individuals may not be as great in a country with more lenient measures. Additionally, on top of social distancing measures, the first few months of the pandemic bore an added “shock factor” which should be taken into account when discussing the impact of the pandemic as things were still relatively new and unpredictable during this phase. Taken together, the mental health and life satisfaction of emerging adults may have been affected by the pandemic more than any other age group and there is evidence to suggest that this impact may differ based on social withdrawal subtypes.

#### Identity Development

In addition to mental distress and life satisfaction, the COVID-19 pandemic may have also affected the identity development of young adults. The formation of one’s identity is one of the key developmental tasks of emerging adulthood (Arnett, 2010) and requires time for identity exploration (Marcia, 1980). However, much of this exploration happens in social contexts. When social opportunities are limited, by external factors like the COVID-19 pandemic, it is possible that identity exploration may also be delayed or inhibited (Gruber et al., 2020). Researchers have hypothesized that these difficulties with identity development may be due to the fact that withdrawing from social interactions means missing out on critical exploration opportunities (Nelson & Millett, 2021).

Indeed, previous work on social withdrawal and identity development has shown that both shy and avoidant emerging adults experience delays in identity development while unsocial emerging adults have been shown to have higher levels of identity exploration than shy and avoidant emerging adults (Schwartz et al., under review) and higher identity commitment than even non-withdrawn emerging adults (Barry et al., 2013). Although the COVID-19 pandemic has limited critical identity exploration opportunities for all emerging adults, we might expect to see a similar delay in identity development during the pandemic for all emerging adults (including non-withdrawn emerging adults) rather than the typical differences across groups. However, unsocial emerging adults may be the exception to this delay. In addition to exploration opportunities, the formation of one’s identity often requires introspection and mentalizing processes that occur in moments of solitude (Paulus et al., 2021). Researchers have suggested that unsocial individuals may be particular skilled at these mentalizing processes, explaining their higher identity commitment than even non-withdrawn individuals (Barry et al., 2013). Thus, we would expect a delay in identity development for shy, avoidant, and non-withdrawn emerging adults, but not for unsocial emerging adults.

### The Current Study

The purpose of this study was to determine whether shy, unsocial, avoidant, and non-withdrawn emerging adults were impacted differently by the COVID-19 pandemic in terms of (changes in) mental distress, life-satisfaction, and identity development. Regarding mental distress, we hypothesized that mental distress would be significantly higher during the pandemic for non-withdrawn individuals and lower during the pandemic for avoidant individuals when compared to pre-pandemic levels. We also hypothesized that there would be no difference in mental distress over time for shy and unsocial individuals, but that shy emerging adults would still report higher levels of mental distress than non-withdrawn and unsocial individuals. Our hypothesis for life satisfaction was similar, in that, we expect non-withdrawn individuals to report lower life satisfaction during the pandemic than before the pandemic, avoidant individuals to report higher life satisfaction during the pandemic, and shy and unsocial individuals to report no change in life satisfaction. With regards to identity development, we expect to see a delay in identity development for shy, avoidant, and non-withdrawn emerging adults but not for unsocial emerging adults.

Finally, recent literature on subtypes of social withdrawal has emphasized the importance of examining a ‘mixed-withdrawn’ group in comparison to the other groups which is comprised of individuals who score high on multiple subtypes of withdrawal (Nelson et al., 2021). Thus, this mixed-withdrawn group will be included in our analyses. However, because relatively little is known about this group, its inclusion is exploratory, and we do not formulate any specific hypotheses about how this group differs from the other subtypes.

## Method

### Participants and Procedure

The participants for this study were part of Wave III and IV of the *Researching Emerging Adults Developmental Years* (READY) study, a nationally representative longitudinal study of emerging adults that began in 2017 (ready.byu.edu). Participants were recruited using a simple random sampling approach of 18 year-olds in the United States, stratified by gender, racial and ethnic distributions, and other demographic characteristics. The sample was identified by and administered an online survey by Qualtrics which collects panel data from their panel respondent list. Once identified, participants were contacted either by mail, email, or telephone (including text messages), and screened for eligibility and interest. After screening was complete they were given a link to complete the online survey. The first page of the survey contained information about informed consent, and participants could not proceed without having given consent. Additionally, this project was reviewed and approved by the IRB of the funding university. The retention rate of participants from wave I to wave III was 58.2%. However, as females were more likely to be retained than males, a purposive sample of 272 men were introduced into the study between waves II and III. Retention from wave III to wave IV was 100%, but participants from previous waves were recontacted at each wave so this retention did not necessarily include the exact same participants from wave III.

Wave III took place from July of 2019 to March of 2020. Wave IV took place from July to December of 2020, during the COVID-19 pandemic. Participants were included if they completed the measures in both waves. Moreover, 71 participants who completed the Wave III survey after March 1^st^ 2020 were excluded to be sure that all data used from this wave was from before the beginning of the pandemic in the United States (Cucinotta & Vanelli, 2020).

In total, 1249 emerging adults were included in the study (37.6% male, 60.3% female, 2.1% other). The average age for participants was 20.5 (SD = .50) at Wave III and all participants were between 20 and 21 years old. In terms of ethnicity, 44.2% of individuals were of European American descent, 10.3% were African American, 16.3% were Latino, 6.5% were Asian American, 2% were biracial, and 1% were of another race. In terms of education, 3.7% had already obtained a college degree, while 51.2% were currently enrolled in college. Fifty percent of individuals were living on their own (not with parents) before the beginning of the pandemic, and 49.8% were living on their own during the pandemic.

### Measures

#### Social Withdrawal (Wave III)

Social withdrawal was measured before the pandemic using ten items from the Child Social Preference Scale (Coplan et al., 2004) adapted for college students (Nelson, 2013). Participants responded on a scale from 1 (*Strongly Disagree)* to 5 (*Strongly Agree*) how much the agreed with statements about shyness (i.e. “I’d like to hang out with other people, but I’m sometimes nervous to”; α = .85), unsociability (i.e. “I’m just as happy to be by myself as with other people”; α = .70) and avoidance (i.e. “I try to avoid social occasions”; α = .75). Higher scores represented higher motivations for social withdrawal. Groups were then formed based on participant’s scores for each type of social withdrawal.

Those who scored higher than one standard deviation above the mean on shyness, but not on unsociability and avoidance characterized the shy group (n = 159; 12.6%). Individuals who scored high on unsociability but not shyness and avoidance characterized the unsocial group (N = 86; 6.9%). Individuals who scored high on avoidance but not shyness and unsociability characterized the avoidant group (N = 112; 9%). Those who scored below one standard deviation above the mean on all three subtypes were considered the non-withdrawn group (N = 739; 59.6%). Finally, those who scored above one standard deviation above the mean on two or more types of social withdrawal characterized a fifth mixed-withdrawal group (N = 143; 11.5%). Of the mixed group 20 scored high on both shyness and unsociability, 55 scored high on both shyness and avoidance, 56 scored high on both unsociability and avoidance, and 27 scored high on all three subtypes of withdrawal.

#### COVID-19 Impact (Wave IV)

The degree to which a person’s life was impacted by the pandemic was measured at Wave IV by asking participants to rate how much the COVID-19 pandemic has influenced their daily life on a scale from 1 to 10 with 10 indicating high COVID-19 impact.

#### Mental Distress (Wave III and IV)

Mental distress was measured both before and during the pandemic using 12 items adapted from Rosenberg (1965) that asks respondents to rate how often they experience a variety of emotions on a scale from 1 (*Never*) to 5 (*Very often*) on questions such as “I feel I am sad and blue” and “I fear losing control of my surroundings.” The twelve items were averaged to form a single scale (α = .93) where higher scores indicated more general mental distress.

#### Life Satisfaction (Wave III and IV)

Life satisfaction was also measured before and during the pandemic using six items created by the principle investigators for the purpose of the project in which respondents rated the extent that they were satisfied with their overall life, family life, social life, financial situation, community, and current job. Responses ranged from 1 (*Very dissatisfied*) to 4 (*Very satisfied*) and were averaged to form a single scale (α = .86) where higher scores represented more life satisfaction.

#### Identity Development (Wave III and IV)

Identity development was measured before and during the pandemic using 12 items adapted from the Dimensions of Identity Development scale (Luyckx et al., 2006). Respondents were asked to rate their agreement on a scale from 1 (*Strongly disagree*) to 5 (*Strongly agree*) on questions such as “My future plans give me self-confidence” and “I have a clear view on my future.” The twelve items were averaged to form a single scale (α = .95) where higher scores indicated greater identity development.

## Results

### Descriptive Statistics

Descriptive statistics for all outcome variables are reported in Table 1. Additionally, paired sample t-tests were conducted to test whether there were differences in the mean level of shyness, unsociability, and avoidance from before to after the pandemic. There were no significant mean differences in shyness (*t*(1237) = 1.32, *p* = .186), unsociability (*t*(1237) = 1.61, *p* = .107), or avoidance (*t*(1237) = −.09, *p* = .929) across time. An ANOVA was also initially conducted to determine whether the social withdrawal groups differed in how much the pandemic had generally impacted their daily lives. Results showed there were no statistically significant differences across groups (*F*(4, 1231) = 1.57, *p* = .181).

### Main Analyses

Three two-way mixed ANOVAs were estimated to determine whether the change in mental distress, life satisfaction, and identity development differed as a function of social withdrawal subgroups before and during the pandemic. For each outcome variable, we examined whether there were mean-level group differences (shy, unsocial, avoidant, mixed-withdrawn, non-withdrawn) or differences across time (from pre-COVID to during COVID), and we tested the group by time interaction to examine whether there were differences over time across groups. Means and standard deviations of the social withdrawal groups are reported in Table 2.

#### Mental Distress

**Figure 1.**
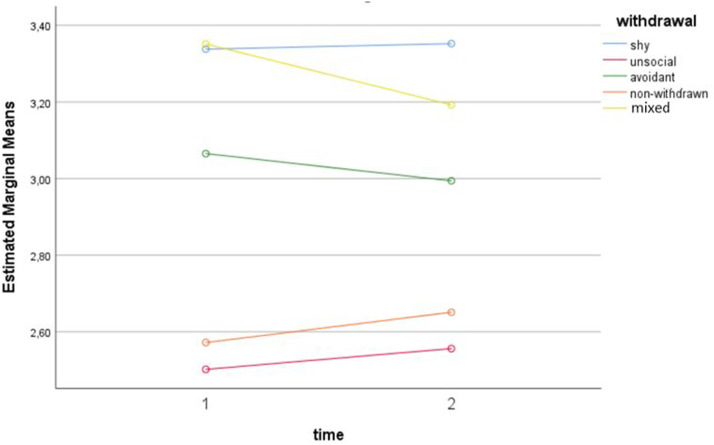
Estimated marginal means of mental distress over time divided by group. *Note*. Change over time was only significant for the non-withdrawn and mixed-withdrawn groups.

Results for mental distress showed a significant main effect for social withdrawal groups (*F*(4, 1234) = 43.009, *p* < .001, partial ƞ^2^ = .122), a non-significant main effect of time (*F*(1, 1234) = 1.000, *p* = .317, partial ƞ^2^ = .001), and a statistically significant group by time interaction (*F*(4, 1234) = 4.817, *p* = .001; partial ƞ^2^ = .015; see Figure 1). In order to interpret this interaction, post-hoc tests of pairwise comparisons with Bonferroni corrections were examined to determine differences between groups at each time point. Additionally, separate univariate analyses were estimated to test differences across time for each group. These analyses showed that unsociable and non-withdrawn individuals reported the lowest levels of mental distress before and during the pandemic, but the non-withdrawn group significantly increased in mental distress across time (*t*(738) = −3.26, *p* = .001) while the unsocial group did not (*t*(85) = −0.78, *p* = .437). The mixed-withdrawn and shy groups reported the highest levels of mental distress before and during the pandemic, but the mixed-withdrawn group significantly decreased in mental distress across time (*t*(142) = 2.72, *p* = .007) while the shy group did not (*t*(158) = 0.29, *p* = .775). The avoidant group also did not significantly decrease in mental distress across time (*t*(111) = 1.04, *p* = .302).

#### Life Satisfaction

The results involving life satisfaction detected a significant main effect for social withdrawal group (*F*(4, 1229) = 37.50, *p* < .001; partial ƞ^2^ = .109), a significant main effect for time (*F*(1, 1229) = 11.550, *p* = .001; partial ƞ^2^ = .009), and a non-significant group by time interaction (*F*(4, 1229) = .919, *p* = .452; partial ƞ^2^ = .003). The main effect of time indicates that life satisfaction during COVID (*M* = 2.61, *SD* = .61) was lower than before COVID (*M* = 2.68, *SD* = .60). To interpret the main effect of social withdrawal groups, pairwise comparisons with Bonferroni corrections were performed. These revealed that the shy, avoidant, and mixed-withdrawn groups reported lower life satisfaction compared to the unsocial and non-withdrawn groups. The shy, avoidant, and mixed-withdrawn groups did not significantly differ from one another, nor did the unsocial and non-withdrawn groups significantly differ from one another.

#### Identity Development

The results involving identity development revealed a significant main effect for social withdrawal group (*F*(4, 1234) = 11.72, *p* < .001, partial ƞ^2^ = .037), a non-significant main effect for time (*F*(1, 1234), = 1.89, *p* = .168, partial ƞ^2^ = .002), and a non-significant group by time interaction (*F*(4, 1234) = .73, *p* = .571; partial ƞ^2^ = .002). Pairwise comparisons with Bonferroni corrections were performed to interpret the main effect of group. These indicated that the shy, avoidant, and mixed-withdrawn individuals reported significantly lower identity development than the unsocial and non-withdrawn individuals. The shy, avoidant, and mixed-withdrawn groups did not significantly differ from one another, nor did the unsocial and non-withdrawn groups significantly differ from one another.

## Discussion

The goal of this study was to determine whether shy, unsocial, avoidant, and non-withdrawn emerging adults were impacted differently by the COVID-19 pandemic in terms of mental distress, life-satisfaction, and identity development over time. We also explored whether these groups were impacted differently than a mixed-withdrawn group who scored high on multiple subtypes of withdrawal. Contrary to our hypotheses, results showed that there were no statistically significant differences between groups in life satisfaction or identity development across time (pre-pandemic to during the pandemic). However, there was a general decrease in life satisfaction over time, irrespective of an individual’s social withdrawal group. Moreover, although identity tends to continue to develop in emerging adulthood (Luyckx et al., 2006), this expected increase was not found in this study. Finally, as hypothesized, non-withdrawn emerging adults reported higher mental distress during COVID than before COVID, while there were no significant differences for shy and unsocial emerging adults. Contrary to our hypotheses however, avoidant individuals also reported no significant differences in mental distress, while those in the mixed-withdrawn group (the group with arguably the highest levels of social withdrawal) reported significantly lower mental distress from before the pandemic to during the pandemic. Thus, there is evidence to suggest that the mental health of socially withdrawn emerging adults was impacted differently by the pandemic than non-withdrawn emerging adults, and these difference depended on specific subtypes of withdrawal.

### General COVID-19 Impact in Emerging Adulthood

Despite the specific focus on emerging adults’ mental health and development, these findings contribute to the broader and expanding literature on the impact of the pandemic on people’s mental health and well-being. Our finding that there were no differences in the broader sample in levels of mental distress from before the pandemic to during the pandemic (July-December of 2020) follows broader trends that show that symptoms of mental distress may have generally returned to pre-pandemic levels after the initial months of the pandemic Robinson et al., 2022). Additionally, it builds off of this work by showing that this may still have been the case after the summer of 2020 when the second wave of the pandemic began and social distancing measures were reinstalled. This is particularly relevant when we consider that emerging adults (more than any other age group) reported the sharpest increases in mental distress during the initial months of the pandemic (Daly et al., 2020; Pierce et al., 2020; Varma et al., 2021) and thus, they may have been the group expected to be most at risk of experiencing lasting changes as well (Haikalis et al., 2021). However, this was not the case. Therefore, this study specifically adds to the literature on the impact of the pandemic by showing that the mental distress of emerging adults in particular may have returned to expected levels even after the summer of 2020. Future research is needed to understand whether this pattern still continued into 2021 or whether there were later changes in mental distress due to the continued strain of the pandemic. It should be noted that our sample includes American participants, and thus, findings may be different in countries that experienced stricter second lockdowns during the winter of 2020-2021 than the United States.

This study also extends previous studies, by looking at the impact of COVID-19 on emerging adults’ life satisfaction and identity development, above and beyond mental health. Indeed, this study shows that emerging adults may have still experienced other long-term effects of the pandemic more unique to their stage of life, even if levels of mental distress may have returned to normal after the initial months of the pandemic. The decrease in life satisfaction is consistent with a number of other studies showing that emerging adults were generally less satisfied with their lives during the pandemic than before (Ammar et al., 2020; Preetz et al., 2021). However, our finding that identity development did not change across time is the first of our knowledge to give evidence to Gruber and colleagues claim (2020) that missed social/academic opportunities and the return of many to living with parents may have led to a stalling or regression of key developmental milestones.

In other words, many young people use college campuses, internships, travel, and employment to explore their identity but these types of settings used for exploration were seriously limited during the pandemic. Thus, many young people had fewer opportunities to explore one’s identity which, as reflected in our results, appears to have hindered their identity development. Future work is necessary to determine which components of identity development may have been specifically affected (i.e., exploration in depth, exploration in breadth, commitment) and whether emerging adults have been able to “catch-up” on their identity development after the return to normalcy, or whether this delay could be associated with delays in reaching other developmental milestones.

### COVID-19 Impact on Socially Withdrawn Emerging Adults

Despite general trends showing that the mental health of emerging adults recovered after the initial months of the pandemic, we know that not all emerging adults follow this trend and there are some groups of individuals who may have been impacted more than others (Germani et al., 2020; Reyes-Portillo et al., 2022; van den Berg et al., 2021). This study extends in a number of ways work by Xu and colleagues (2022) that examined the impact of the pandemic on socially withdrawn adults in China, by showing that a person’s motivations for approaching and avoiding social interaction *before* the pandemic are factors that should be taken into account when examining the differential impact of the COVID-19 pandemic on certain individuals.

### Mental Distress

In line with our hypotheses, the mental distress of shy individuals remained high and stable from before the pandemic to during the pandemic while it remained low and stable for unsocial individuals. However, contrary to our hypotheses and the work by Xu and colleagues (2022), the mental distress of avoidant individuals also remained high and stable (or at least the small decrease over time was not statistically significant). It has been suggested in the past that avoidant individuals may have a high aversion to social interaction in part because of their elevated levels of mental distress (Coplan et al., 2015). Indeed, avoidant emerging adults score higher on self-harm and suicidal ideation than any other subtype (Nelson, 2013). Thus, as a number of longitudinal studies on the impact of the pandemic have shown that the largest predictor of mental distress during the pandemic was mental distress before the pandemic (Luchetti, 2020; van den Berg et al., 2021), it makes sense that avoidant emerging adults who experienced higher levels of mental distress before the pandemic would still be experiencing mental distress during the pandemic.

Another important extension of what has been found previously (Xu et al., 2022) is that there was a slight increase in mental distress for non-withdrawn emerging adults from before the pandemic to during the pandemic (after the summer of 2020). This is especially notable because it does not match the typical patterns of mental distress that we see in the general population showing that levels of mental distress returned to normal after the initial months of the pandemic (Robinson et al., 2022). Thus, it identifies at least one specific group that may vary from the norm: highly social emerging adults. Indeed, previous literature examining how the pandemic influenced extroverts and introverts showed that highly extroverted individuals increased in loneliness during the initial months of pandemic and decreased in social connectedness (Folk et al., 2020; Liu et al., 2021). This study builds off that work by showing that some of these effects may have lasted even later into the pandemic for emerging adults in particular. Emerging adulthood is arguably the time in life that affords the most autonomy over how and with whom a person spends their time (Nelson & Millett, 2021). This is often accompanied by a shift towards spending more time with peers than ever before (Doumen et al., 2012). Thus, it makes sense that non-withdrawn emerging adults who would normally be using their autonomy to be with peers would struggle when their social opportunities were limited, even in later stages of the pandemic after the initial shock wore off. This may especially be the case when compared to withdrawn emerging adults who may be more “used” to spending time in isolation.

Finally, previous work has failed to examine the impact of the pandemic on those emerging adults who are high in multiple aspects of withdrawal (avoidance, shyness, unsociability). In general, we know very little about the mixed-withdrawn group except that they are the group that scores the highest on indices of social withdrawal. Until recently, this group has been left out of most studies on subtypes of social withdrawal (Nelson & Millett, 2021), and thus their conceptual distinctiveness from the other subtypes has yet to be systematically explored. This is quite surprising as it is a sizeable group of emerging adults (12% of the entire sample). Nevertheless, the fact that at least one group of socially withdrawn individuals *decreased* in mental distress from before the pandemic to during the pandemic is notable as it makes this one of the very few studies that have uncovered a benefit of the pandemic for a segment of the population in, at least, one area of their lives.

Emerging adults who experience extremely high desires to avoid social interaction and/or spend time alone may have experienced fewer symptoms of mental distress during the pandemic than before the pandemic. This is becomes relevant even beyond the pandemic as it suggests that broader societal expectations about sociability may play a key role in how social withdrawal is associated with indices of well-being. A unique byproduct of the pandemic was that social distancing requirements temporarily lowered (if not eliminated) societal pressures to socialize for a brief moment in time. Our findings suggest that there were at least some individuals who benefited from that shift in expectations. Though the pandemic has subsided and social expectations may have returned to normal, future work may examine whether there are other ways to help highly socially withdrawn individuals lower and/or manage cultural and societal pressures to be social in a way that would benefit them. Additionally, future work is needed to determine how scoring high on multiple types of social withdrawal may be associated with different correlates and consequences than scoring high on just one type of withdrawal irrespective of the COVID-19 pandemic.

### Life Satisfaction and Identity Development

The life satisfaction and identity development of emerging adults did not differentially change across times as a function of social withdrawal subtype. However, as expected, shy, avoidant, and mixed-withdrawn individuals reported lower life satisfaction and identity development than unsocial and non-withdrawn individuals before and during the pandemic. Thus, all groups decreased in life satisfaction despite initial mean-level differences, and all groups did not change with regards to identity development. Originally, we had hypothesized that we might not see a delay in identity development for unsocial emerging adults as they may be particularly skilled at the mentalizing processes required for identity formation (Paulus et al., 2021). However, since unsocial emerging adults only score higher than others on identity *commitment* and not identity *exploration* (Barry et al., 2013), it may be that these mentalizing skills aid more in the process of committing to one’s identity than exploring it. Thus, as exploration typically comes before commitment, and as the pandemic limited exploration opportunities for all emerging adults regardless of social withdrawal, unsocial adults were equally affected.

### Limitations and Conclusion

Though this study has a number of strengths (representative sample, longitudinal nature, etc.) there are also a few limitations that should be mentioned. First, this study did not examine multiple assessments during the initial stage and peak of the pandemic in the spring of 2020. As such, we were not able to say anything about differences across groups during this key time period or examine potential differences with our current assessments tapping into the continued impact of the pandemic. Additionally, social withdrawal subgroups were formed using a person-centered approach that has been used across a number of studies (e.g., Nelson, 2013; Nelson et al., 2016), but the cut-off points used to distinguish the social withdrawal groups are sample dependent. This method was used as it matches current theories regarding social withdrawal (Asendorpf, 1990) and because it allows us to better compare and interpret our findings against the backdrop of existing literature that also examines subtypes of social withdrawal in this way. However, future research should also explore ways to study subtypes of social withdrawal using a method that creates groups in a more statistically sophisticated manner.

In conclusion, nearly everyone has experienced some of the effects of the COVID-19 pandemic, but emerging adults seem to be especially affected. In addition to a decrease in life satisfaction, this study shows a potential delay of identity development during the pandemic, a key developmental milestone of emerging adulthood. Finally, not all emerging adults may be equally affected as non-withdrawn emerging adults experienced increases in mental distress while extremely withdrawn individuals experienced a decrease in mental distress. Thus, this paper suggests that individual factors should be taken into account in attempts to better understand the impact of the COVID-19 pandemic on emerging adults. Additionally, it gives further evidence that social withdrawal is an important factor in explaining individual differences in mental health and social development during a stage of life when social interaction is key in achieving many milestones.

## Supplemental Material

Supplemental Material - Perks of the Pandemic?: The Impact of the COVID-19 Pandemic on Socially Withdrawn Emerging Adults Click here for additional data file. Supplemental Material for Perks of the Pandemic?: The Impact of the COVID-19 Pandemic on Socially Withdrawn Emerging Adults by Mallory A. Millett, Larry J. Nelson, William J. Burk, and Yvonne H. M. van den Berg in Emerging Adulthood

## Appendix

**Table 1.** Bivariate correlations and descriptive statistics for all outcome variables.

	1	2	3	4	5	6
1. Mental Distress (Pre-pandemic)	—					
2. Life Satisfaction (Pre-pandemic)	−.67***	—				
3. Identity Development (Pre-Pandemic)	−.38***	.50***	—			
4. Mental Distress (Pandemic)	.74***	−.54***	−.36***	—		
5. Life Satisfaction (Pandemic)	−.55***	.69***	.41***	−.70***	—	
6. Identity Development (Pandemic)	−.36***	.41***	.61***	−.45***	.57***	—
*M/SD*	2.79 (.96)	2.68 (.60)	3.43 (1.09)	2.83 (.95)	2.61 (.61)	3.37 (1.09)
Range	1-5	1-4	1-5	1-5	1-4	1-5

*Note.* ****p* < .001, ***p* < .01, **p* < .05.

**Table 2.** Means and standard deviations from two-way mixed ANOVAs.

	Mental Distress				Life Satisfaction				Identity Development			
	Pre-COVID		During-COVID		Pre-COVID		During-COVID		Pre-COVID		During-COVID	
	M	SD	M	SD	M	SD	M	SD	M	SD	M	SD
Shy	3.33^ac^	.89	3.34^a^	.88	2.40^a^	.58	2.35^a^	.60	3.20^a^	1.23	3.03^a^	1.19
Unsocial	2.50^b^	.90	2.56^b^	.95	2.91^b^	.61	2.80^b^	.62	3.67^b^	1.12	3.59^b^	1.05
Avoidant	3.06^a^	.90	3.00^c^	.90	2.44^a^	.62	2.38^a^	.56	3.17^a^	1.09	3.20^a^	1.11
Mixed-withdrawn	**3.36** ^**c**^	1.07	**3.21** ^**ac**^	1.06	2.43^a^	.66	2.42^a^	.66	3.15^a^	1.26	3.16^a^	1.31
Non-Withdrawn	**2.56** ^**b**^	.86	**2.66** ^**b**^	.87	2.80^b^	.52	2.73^b^	.57	3.56^b^	.98	3.50^b^	.99

*Note.* differing superscripts in a column indicate significant differences across groups. Bolded numbers in a row indicate significant differences across time.
