# Supplementary material for: Perks of the Pandemic?: The Impact of the COVID-19 Pandemic on Socially Withdrawn Emerging Adults
Source: Emerg Adulthood. 2023 Jun 26:21676968231184283. doi: 10.1177/21676968231184283 (PMC10293869; doi:10.1177/21676968231184283)
Supplement: Supplemental Material - Perks of the Pandemic?: The Impact of the COVID-19 Pandemic on Socially Withdrawn Emerging Adults [file sj-pdf-1-eax-10.1177_21676968231184283.pdf]

Emerging Adulthood
Open Practices Disclosure Form and Badge Application

Manuscript Title:___Perks of the Pandemic?: The Impact of the COVID-19 Pandemic on Socially Withdrawn Emerging Adults___

Manuscript #: _ EA-22-0222.R1__ **Corresponding Author:___Mallory A. Millett ___**

Articles accepted to *Emerging Adulthood* after September 1, 2018 are eligible to earn badges that recognize open scientific practices: publicly available data, material, or preregistered research plans. Please read more about the badges on the journal home page, and you can also find information on the Open Science Framework wiki.

Please check one of the following boxes:

[ ] I am interested in applying for a badge and have included the necessary information below.

[x] I am not interested in applying for a badge (please sign at the end of the form and return to the production office)

To apply for one or more badges acknowledging open practices, please check the box(es) corresponding to the desired badge(s) below and provide the information requested in the relevant sections. To qualify for a badge, you must provide a URL, doi, or other permanent path for accessing the specified information in a public, open-access repository. **Qualifying public, open-access repositories are committed to preserving data, materials, and/or registered analysis plans and keeping them publicly accessible via the web in perpetuity.** Examples include the Open Science Framework (OSF) and the various Dataverse networks. Hundreds of other qualifying data/materials repositories are listed at http://re3data.org/. Preregistration of an analysis plan must take place via a publicly accessible registry system (e.g., OSF, ClinicalTrials.gov or other trial registries in the WHO Registry Network, institutional registration systems). **Personal websites and most departmental websites do not qualify as repositories.**

Authors who wish to publicly post third-party material in their data, materials, or preregistration plan must have the proper authority or permission agreement in order to do so.

There are circumstances in which it is not possible or advisable to share any or all data, materials, or a research plan publicly. For example, there are cases in which sharing participants' data could violate confidentiality. If you would like your article to include an explanation of such circumstances and/or provide links to any data or materials you have made available—even if not under conditions eligible to earn a badge—you should do so as part of the Transparency and Openness Statement that must be included with your article.

[ ] Open Data Badge

1. Provide the URL, doi, or other **permanent path** for accessing the data in a **public, open-access repository**:

   [ ] Confirm that there is sufficient information for an independent researcher to reproduce **all of the reported results**, including codebook if relevant.

[ ] Open Materials Badge

1. Provide the URL, doi, or other **permanent path** for accessing the materials in a **public, open-access repository**:

   [ ] Confirm that there is sufficient information for an independent researcher to reproduce **all of the reported methodology**.

[ ] Preregistered Badge

1. Provide the URL, doi, or other **permanent path** to the registration in a **public, open-access repository**:

2. Was the analysis plan registered prior to examination of the data or observing the outcomes? Please note, *Emerging Adulthood* only issues pre-registration badges for manuscripts that contain pre-registered analyses.

3. Were there additional registrations for the study other than the one reported? If yes, provide links and explain.

4. Were there any changes to the preregistered analysis plan for the primary confirmatory analysis? If yes, explain.

5. Are all of the analyses described in the registered plan reported in the article? If no, explain.

No badge will be awarded if (1) is not provided and/or (2) is answered “no,” **or** if (3) is answered “yes” without strong justification, **or** if (5) is answered “no” without strong justification.

By signing below, authors affirm that the above information is accurate and complete, that any third-party material has been reproduced or otherwise made available only with the permission of the original author or copyright holder, and that publicly posted data do not contain information that would allow individuals to be identified without consent.

Signature: Mallory Millett ______________________ **Date: 05 June 2023 _**

Name:___Mallory Millett____________________________

This form is based on a template provided by the Open Science Framework, https://osf.io/5fndw/
